# Identifying Meibomian Gland Dysfunction Biomarkers in a Cohort of Patients Affected by DM Type II

**DOI:** 10.3390/vision7020028

**Published:** 2023-03-24

**Authors:** Alessandro Abbouda, Antonio Florido, Filippo Avogaro, John Bladen, Enzo Maria Vingolo

**Affiliations:** 1 Department of Ophthalmology, Alfredo Fiorini Hospital, 04019 Terracina, Italy; 2 Department of Biotechnology and Medical-Surgical Sciences, “Sapienza” University of Rome, 04100 Latina, Italy; 3 Oculoplastic Department King’s College Hospital NHS Foundation Trust, Denmark Hill, London SE5 9RS, UK

**Keywords:** mibomian gland dysfunction, diabetes mellitus, hormones, biomarkers

## Abstract

(1) Background: Meibomian gland dysfunction (MGD) among patients with diabetes mellitus (DM) is a common manifestation of dry eye syndrome (DES). (2) Methods: The purpose of this study is to identify clinical parameters and biomarkers useful to improve the follow-up and the treatment of these patients. We have used an ocular surface disease index (OSDI) questionnaire, Schirmer test I/II, tear film break-up time (TF-BUT), fluorescein plus lissamine green staining, Marx’s line (ML), and meibomian gland (MGs) morphology using Sirius^®^ Topographer (CSO, Costruzione Strumenti Oftalmici, Florence, Italy). Blood sample analysis included glucose, glycated hemoglobin, lipid profile, cortisol, dehydroepiandrosterone sulfate (DHEA-S), androstenedione (ASD) and testosterone. (3) Results: Cortisol and ASD were positively correlated with an increase of MG tortuosity, and an Increased level of triglycerides was associated with a reduction of MGs length. DHEAS levels lowered with age and were associated with ocular surface staining. (4) Conclusions: Future studies, perhaps including meibum lipid analysis and tear cytokine levels, may also further elucidate the connection between these parameters, MG architecture and function.

## 1. Introduction

Meibomian glands (MGs) are modified sebaceous glands within the upper and lower tarsal plate and are distributed vertically to the eyelid margin. MGs are more abundant in the upper eyelids and are longer, storing twice the amount of lipid compared to the lower eyelid MGs [1]. Lipid secreted by MGs is termed meibum which is released from their visible orifices just posterior to the mucocutaneous junction along the eyelid margin. Meibum plays an key role in tear film stability, reducing the evaporation of the aqueous layer and protecting the eye surface as a hydrophobic barrier [1].

The impairment of MGs leads to meibomian gland dysfunction (MGD), a chronic inflammation resulting in terminal duct occlusion, and modified lipid secretion. MGD is the most common reason for evaporative dry eye syndrome (DES). Patients often suffer from protracted dryness, and burning and foreign body sensation, and can experience compromised quality of life [2].

Numerous studies have suggested a connection between MGD and systemic factors, such as androgen deficiency [3], hyperlipidemia [4] and diabetes mellitus (DM) [5]. It has recently been found that DM is a significant risk factor for the development of MGD [4]. The association between DM and the risk of development of DES is also supported by a recent meta-analysis that considered over 2.5 million patients from a total of 17 studies [6]. This association has a pathogenetic rationale considering that the MG is a modified sebaceous gland and that sebaceous gland structure and function are altered in diabetic subjects [4,5]. It has been hypothesized that altered insulin production/response and hyperglycemia are key elements in the pathogenesis of MGD in diabetic subjects. Insulin is fundamental for a correct sebaceous gland metabolism; deficiency of insulin would promote dysfunction [5]. In addition, hyperglycemia is a stimulus for lipolysis in adipocytes and this response in MGs can reduce the quality of meibum [7]. These pathogenetic mechanisms are supported by recent evidence showing that insulin is able to stimulate the proliferation of immortalized human meibomian gland epithelial cells (HMGECs), whereas high glucose was found to be toxic for HMGECs [5]. MGD and associated evaporative tear loss is followed by an inflammation increase on the eye surface with the growing of bacterial populations supported by abnormal lipids as substrates [8]. It has also been found recently that the chronic inflammatory status in patients with MGD is related to high concentrations of inflammatory cytokines in the tear film [9,10]. In our study, we look at serum markers and the development of MGD in DM patients.

## 2. Material and Methods

This cross-sectional study included patients with type II DM and symptoms of MGD attending the Department of Ophthalmology at A. Fiorini Hospital in Terracina (Latina, Italy) in the last six months. The study was performed in conformity with the principles of the Declaration of Helsinki, the International Conference on Harmonisation and the Good Clinical Practice recommendations. Informed consent was acquired from all subjects prior to the start of the study. Inclusion criteria were patients affected by DM type II with symptoms of MGD. Exclusion criteria included: recent ocular infections, seasonal allergies, any history of ocular surgery, use of any medications or eye drops known to affect the ocular surface, a current or prior contact lens wearer, pregnant by self-report, thyroid disease, autoimmune disease, contraceptive use, smokers and anti-androgen and menopause hormonal replacement therapy.

All subjects were requested to complete the Ocular Surface Disease Index (OSDI) Allergan, Inc., Irvine, CA, USA. Questionnaire [11]. Briefly, the patients mark the intensity of their symptoms on a scale of 0–4 (0: none of the time, 1: some of the time, 2: half of the time, 3: most of the time, 4: all of the time). The final score was measured and ranged from 0 to 100 (score 0–12: normal, 13–22: mild dry eye disease, 23–32: moderate dry eye disease, greater than 33: severe dry eye disease).

Biomicroscopic anterior segment examination occurred and the eyelid margin was analysed for presence of telangiectasia, lid margin abnormalities, occluded MGs orifices and anterior/posterior displacement of the mucocutaneous junction. The latter was assessed according to Yamaguchi et al. [12]. A Marx line (ML) score is reported in Table 1.

Tear film break-up time (TFBUT) was determined as follows: subjects were asked to blink 3–4 times after fluorescein instillation into the lower fornix. TFBUT was assessed by the time between the last blink and the appearance of the first black spot; the results of three measurements were registered. [13] Ocular surface staining using lissamine green was used to determine the Van Bijsterveld score (vBS): the ocular surface is divided into three areas; nasal bulbar conjunctiva, temporal bulbar conjunctiva and cornea. Each area is evaluated on a scale of 0 to 3, with 0 indicating no staining and 3 indicating confluent staining; the maximum reachable score with this system is 9 [13]. The Schirmer test evaluates aqueous tear production and is performed using sterile paper strips applied into the inferior-temporal side of the conjunctival sac of both eyes. The wetted length, in millimeters, is measured after 5 min. There are two versions of the test: without topical anesthesia to assess the total tear secretion, which is the sum of reflex and basal tear flow (Schirmer test I), and with topical anesthesia to quantify basal tear secretion (Schirmer test II) [14]. Only data from the same eye where meibography was performed were included in the results.

**Figure 1:**
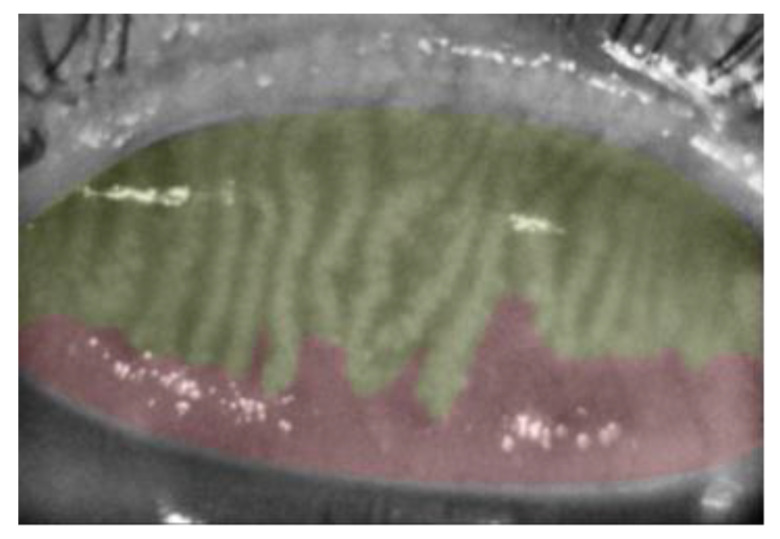
Meiboscore Grade 2. Meibomian gland area is highlighted in yellow. The area of meibomian gland loss (red) is 33.4%.

Meibography was performed on one randomly selected eye of each subject using the Sirius^®^ Topographer (CSO, Costruzione Strumenti Oftalmici, Florence, Italy) [15]. Only upper eyelids were analyzed. Three images for each eye were recorded. The device determines the dropout area semi-automatically by percentage and assesses meiboscore as follows: grade 0, no loss at all; grade 1¼, ≤25%; grade 2, 26–50%; grade 3, 51–75%; and grade 4, >75% (Figure 1).

### 2.1. Image Analysis

**Figure 2:**
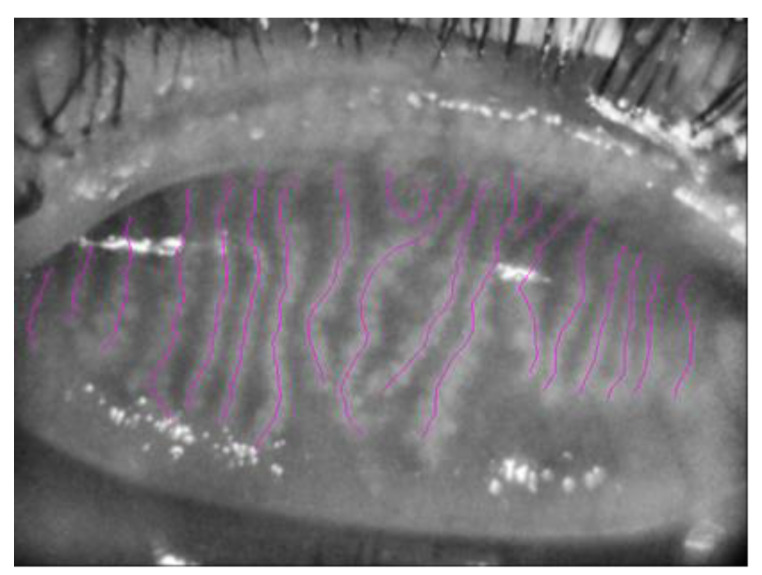
Meibomian Glands highlighted in purple using NeuroJ Tracing tool.

The three best focused representative images of one random eye, with good image brightness and contrast, were selected for MGs length, width and area analysis. Two masked observers (AF and FA) performed the meibography images analysis. Briefly, MG length was quantified using NeuronJ (http://www.imagescience.org/meijering/software/neuronj/, (accessed on 10 October 2022)), a plug-in of ImageJ (http://www.imagescience.org/meijering/software, (accessed on 10 October 2022)) that was used previously for semi-automated corneal nerve tracing [16] but can also be useful to quantify MG length and width (Figure 2).

**Figure 3:**
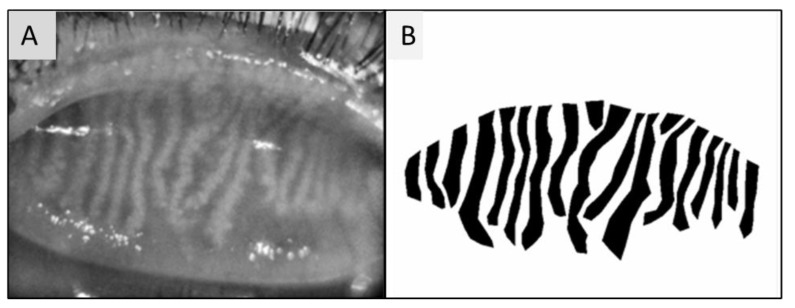
(**A**) Upper eyelid meibography. Image taken by Sirius^®^ Topographer (CSO, Costruzione Strumenti Oftalmici, Florence, Italy). (**B**) Image processed by Image J, obtained from polygonal boundaries traced around meibomian glands.

Main MGs were defined as total number of MGs in one image; MG lengths were MG length/frame area converted to mm/mm^2^. MG width was calculated at the top, centre and bottom of each gland and reported as MG width/frame area converted to mm/mm^2^. The total MG number and MG length and width were calculated by summing the main MGs, length and width, respectively. MG area was analyzed using the previous method reported [15,17] but differently from this method, we set the exterior image to white, and the interior to black in order to generate a binary ground truth mask. A polygon border around the glands was created and saved. The value was reported as area in mm^2^ (Figure 3).

A fasting blood sample was collected to analyze glucose levels, glycated haemoglobin (HbA1c) triglycerides, total cholesterol, HDL (high-density lipoprotein) cholesterol, LDL (low-density lipoprotein), dehydroepiandrosterone sulfate (DHEA-S), androstenedione (ASD), cortisol and testosterone level.

### 2.2. Statistical Analysis

Continuous variables were expressed as mean ± standard error of the mean. In terms of Kolmogrov–Smirnov test and Shapiro–Wilk test, our data were approximately normally distributed. *p*-values were above 0.05 for all variables except for the testosterone level. The Spearman correlation was performed to measure the intensity and direction of association between two variables. The one-way ANOVA test was used for comparison of more samples. Linear regression analysis was performed to analyze the biomarkers according to the MG variables obtained using meibography. Regression analysis using a pairwise selection method was performed to identify the most important variable associated to the MGD. Intraclass correlation coefficient (ICC) was calculated between two masked observers for MGs length, width and area. Statistical analysis was realized using SPSS version 22 (SPSS Inc., Chicago, IL, USA) and a *p*-value of less than 0.05 was considered significant.

## 3. Results

One hundred twenty-three patients with type II DM with symptoms of MGD attended the department of ophthalmology at A. Fiorini Hospital in Terracina (Latina, Italy). Of these, 69 patients had a recent history of cataract surgery, four had thyroid disease, 22 were heavy smokers, three were taking anti-androgen therapy and seven menopause hormonal replacement therapy; therefore, 18 female patients (14.6%) met the inclusion criteria and were included in the study. Demographics and clinical signs of studied subjects are summarised in Table 2. Seventy percent of patients had a Schirmer test I value < 10 mm/5 min and 94.1% of patients had a Schirmer test II <10 mm/5 min, and 47.1% of patients had a value lower than 4 mm/5 min. TFBUT was lower than 5 s in 94.4% patients. Median value of the ML score was 3 and vBs was 2. OSDI score was higher than 20 in 53.1%; the median value was 28.5. Fifty-six percent of patients were classified as normal weight according to their BMI percentile and 44% were overweight.

### 3.1. Blood Biomarkers

Mean glycemia values were 124.56 ± 16.65 mg/dL (min 88 max 152), and Hb1Ac of 6.5% ± 0.69 (min 5.7%, max 8.3%). Seventy-five percent of patients had a value of Hb1Ac ≤ 6.5%. Mean triglycerides levels were: 145.5 ± 67.9 mg/dL (min 70 max 319); mean total cholesterol was 165 ± 32.59 mg/dL (min 117 max 245); mean HDL value was 45.5 ± 9.15 mg/dL (min 32 max 60); mean LDL was 90.3 ± 20.3 mg/dL (min 54 max 129); mean testosterone level was 1.0 ±1.7 nmol/L (min 0.13 max 5.14); mean DHEA-S value was 122.37 ± 110.52 µg/dL (min 40.5 max 343); mean ASD value was 1.6 ± 0.93 ng/dL (min 0.35 max 3.29); mean cortisol value was 11.54 ± 3.46 µg/dL (min 6.8 max 17.9).

**Figure 4:**
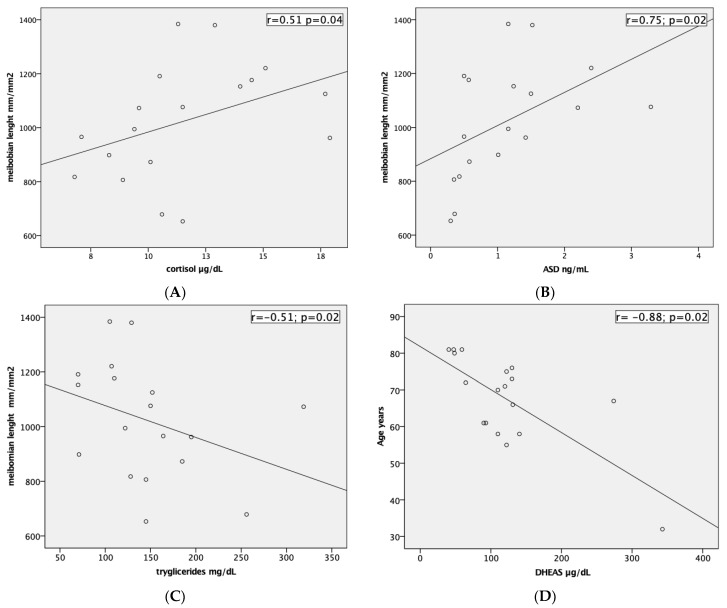
Scatter plots amongst significant variables: (**A**) Serum level of cortisol (µg/dL) and meibomian glands length (mm/mm^2^). (**B**) Serum level of ASD (ng/mL) and meibomian glands length (mm/mm^2^). (**C**) Serum level of triglycerides (mg/dL) and meibomian glands length (mm/mm^2^). (**D**) Serum level of DHEAS (µg/dL) and age (years).

DHEA-S showed a negative correlation with age (r = −0.88; *p* = 0.02), and with the vBS (r = −0.78; *p* = 0.02). Cortisol and ASD were positively correlated with MG length (r = 0.51; *p* = 0.04 and r = 0.75; *p* = 0.02), while triglycerides had a negative correlation (r = −0.51; *p* = 0.02). Scatter plots of the significantly correlated variables are reported in Figure 4.

No significant correlation was found for the other parameters analyzed.

### 3.2. Meibography Analysis

**Figure 5:**
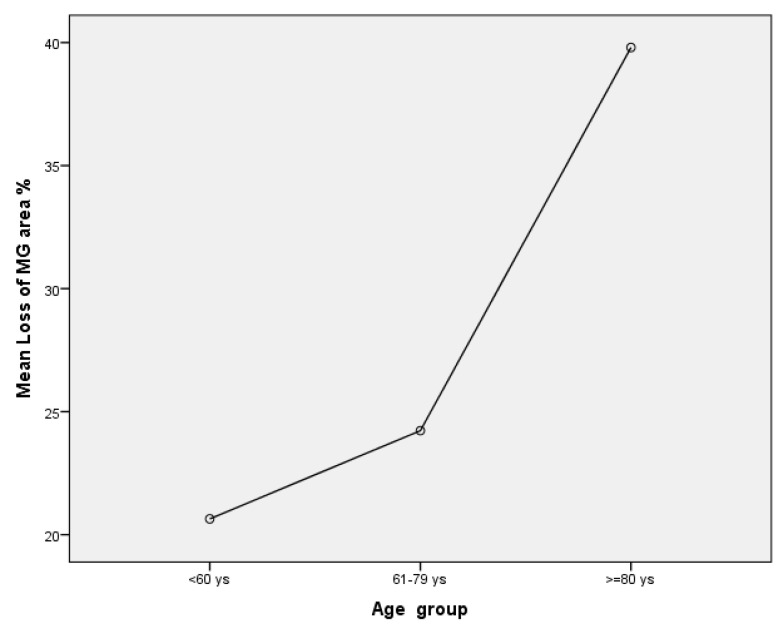
This graph reports mean of loss of MG area amongst groups of different age.

An average of 17.12 ± 2.83 MGs were identified in each eyelid analyzed (min 13–max 18). The mean total MG length was 1023.94 ± 213.07 mm/mm^2^ (min 653 max 1384.) The MG lengths had a negative association to the loss of MG area (r = −0.51; *p* = 0.02). The mean MG width was 75.88 ± 15.53 mm/mm^2^ and was negatively correlated to Schirmer test II and TFBUT (r = −0.5; *p* = 0.02; r = −0.56; *p* = 0.01). The MG area was 172.69 ± 31.29 mm^2^ and was also negatively correlated to Schirmer test II (r= −0.49; *p* = 0.04;). Fifty per cent of patients had a meiboscore value of 2 (loss of 26–50% of MGs) and the other 50% a meiboscore of 1 (loss of ≤25%). Both meiboscore and loss of MG area were positively associated with age (r = 0.55; *p* = 0.01 and r = 0.47; *p* = 0.04). There was a statistically significant difference between loss of MG area and age groups as determined by one-way ANOVA (F = 5.61, *p* =0.015). A Tukey post hoc test revealed that there was a statistically significant difference between the older group and patients younger than 60 years (*p* = 0.01) and the group of patients between 61 and 79 years (*p* = 0.04). However, there were no differences between the groups younger than 60 years and patients between 61–79 years (*p* = 0.75). Mean Loss of MG area was 26.29 ± 11.62% (min 5–max 47). Loss of MG area was higher in the older group (≥80 years; four patients mean value 39.8 ± 6.61%) in comparison to group of patients of 61–79 years (seven patients, mean value 24.23 ± 11.87%) and ≤ 60 years (seven patients, mean value 20.64 ± 7.47%); see Figure 5. There was a high agreement between observers in calculating MG length, width, area and meiboscore (Intraclass correlation coefficient (ICC), respectively, of 0.98; 0.88, 0.96 and 0.94).

Regression analysis was performed between MG parameters obtained by meibography and serum biomarkers. Results are reported in the Table 3.

Regression analysis using a pairwise selection method identified that the age had a significant impact on loss of MG area (F = 5.4; *p* = 0.02). There was a 19.1% difference between the mean of MG loss area of patients older than 80 years and patients younger than 60 years. The difference was statistically significant (IC 95% 4.2–34.1; *p* = 0.01). Similarly, the 15.5% difference between the loss of MG area of patients of 61–79 years old and patients older than 80 years was statistically significant (IC95% 0.67–30.45; *p* = 0.04). In contrast, no statistically significant value was identified for all other analyzed variables.

## 4. Discussion and Conclusions

The purpose of this study was to identify any serum biomarkers in patients affected by type II DM and MGD. Cortisol and ASD were associated with an increase in MG tortuosity and a high level of triglycerides was associated with a reduction of MG length. Moreover, a decay of DHEAS was correlated to a negative modification of ocular surface staining using lissamine green (vBs score) and MG width was negatively correlated with Schirmer test II and TFBUT.

### 4.1. Role of Age and Diabetes

Previous studies have demonstrated how aging is a key factor for the development of MGD [18,19,20] deriving from MG atrophy [21] and a decrease in meibocyte differentiation [19]. Hashemi et al. [20] reported an MGD prevalence of 71.2% in patients older than 60 years. Furthermore, the prevalence gradually increased from 64.4% in the group of 60–64 years to 82.4% in patients older than 80 years [19]. Our paper reported similar finding in that the loss of MG area was more common in those more than 80 years old. In this group of patients, there was a mean loss of MG area equal to 40% compared to a mean of 20% in the younger group. This reduction of MG area can obviously lead to a reduction of meibomium in the tear film and lead to an increase in evaporative DES. However, data obtained from meibography alone cannot discriminate MGD from non-MGD changes as a percentage of MG atrophy is normal amongst older people; thus, morphological and functional MG tests are necessary to determine the presence of MGD.

The association of type II DM and MGD has already been reported in the literature [6,22,23,24,25,26,27,28]. A prospective randomized controlled trial conducted to investigate MG and tear film function in type 2 DM patients found eyelid margin abnormalities to be significantly higher and the number of expressible glands to be significantly lower compared to a control group. Tao Yu et al. [22,26] showed that 57.6% of people in the DM group had MG dropout, while it was 33% in the control group. In addition, they described modifications of MGs, such as enlargement of acinar units, irregular shape with acinus and reduction in density of acinar units. The authors theorized that there may be a certain degree of occlusion of the MG ductal in patients with type 2 DM. The obstruction of the MG ductal leads to accumulation of meibum, thus causing the cystic dilatation and the altered morphology of the acini, and further causing the atrophy of the acini and decrease in acinar density. These findings supported our result of MG loss and modification of ocular surface staining as a reduction of MG acini function.

We also found that MG width was negatively correlated to TFBUT and Schirmer test II value. This means that this MG width can predict the reduction of function of MGs. We hypothesized that if the MG body accumulates meibomium and it is not able to express it through orifices, it can lead to a progressive MG atrophy. This condition is obviously reflected by ocular surface parameters that influenced negatively both the TFBUT and the Schirmer test.

Fan Fang et al. [23] investigated the relationship between MGD and levels of HbA1c in patients with DM type II and reported that HbA1c ≥ 7% is likely to result in MG dysfunctions, especially related to lipid layer thickness and MGs percentage loss. We performed a similar analysis dividing patients according to their level of HbA1c, but we did not identify any differences among variables analyzed. This fact could be related to our small sample but also to a population of diabetics with a good glycemic compensation and low fluctuation of HbA1c. Only five patients had Hba1c values higher than 7%, so comparison between these groups cannot provide useful information. It would be interesting, in order to understand how much age and diabetes affects the MG change, to study a large sample over the years including non-diabetic patients and compare meibography changes according to glycemia value.

### 4.2. Hormones Role

The role of androgens in MG function has been already elucidated [29,30,31]. The MGs are sebaceous glands, and androgens are well known to regulate the development, differentiation and lipid production of sebaceous glands throughout the body [29,32]. Sebaceous gland activity and secretion decrease with age, and this aging-associated dysfunction has been correlated with both atrophy of acinar cells and a reduction in serum androgen levels [33]. Sullivan et al. have demonstrated that aging in men and women goes together with a significant increase in lower eyelid erythema, telangiectasia, keratinization, irregular posterior margins, orifice metaplasia and opaque secretions [34]. Moreover, the assumption of anti-androgen medications leads to MGD, altered lipid profiles in MG secretions, decreased tear film stability and evaporative dry eye [35].

Our population reflected these studies; DHEAS decreased with the age, and it was associated with a modification of ocular surface staining (vBS). The regression analysis between DHEAS and age showed that the age predicts DHEAS level well (F = 17.9; *p* < 0.001), suggesting that 52% of the variation was predicted by the age. The decrease in production of DHEAS with age is considered to play an important role in IL-6-mediated pro-inflammatory effects in humans [36]. DHEAS and ASD concentration dependently inhibited IL-6 production from peripheral blood mononuclear cells but their levels significantly decreased with age and this fact leads to the rise in IL-6 production during the process of aging [36]. This finding could be a significant cofactor for the manifestation of inflammatory and age-related diseases such as MGD. Higher tear levels of interleukin (IL)-1β, IL-6, chemokine IL-8, IL-10, IFN-γ, and tumor necrosis factor-α, TNF-α have been found in dry eye patients [10]; further studies are needed to define if IL-6 in MGD patients can be a good biomarker of this pathology. A high level of cortisol was associated with a higher level of IL-6 and the severity of inflammation was reported to be associated with a low DHEAS/cortisol ratio [37]. Interestingly, we highlighted also that both cortisol and ASD were positively associated with MG length; this result could mean that these hormones are biomarkers of MG length, and that they can have a role in the follow-up of MGD patients. However, only ASD showed a significant result in the regression analysis. ASD predicts a significant effect on the MG length (F = 4.72; *p* < 0.045), suggesting that 22% of the variation is predicted by this factor. This means that it can be considered as a serum biomarker of MGs modification even if a large sample needs to be studied to confirm this theory.

### 4.3. Blood Lipid Levels BMI and Role

A systematic metanalysis reported a strong positive correlation between dyslipidemia and MGD and suggested to perform prospective studies to demonstrate a temporal relationship with MGD preceding dyslipidemia [38]. In our study, we do not identify this association because our population had an overall normal value of total cholesterol and only one patient had a value higher than 200 mg/mL, so the sample was not representative for this condition. Interestingly, a high level of triglycerides was related to a reduction of MG length. Butovich et al. [39] described a new condition that they termed High Triglycerides/Low Waxes (HTLW) syndrome. They observed severely decreased pools of normal meibomian lipids such as wax esters and cholesteryl esters in meibum and tears, and a 20× to 30× rise in the triglyceride fraction over the norm without any change in the routine blood lipid panel test. Our association between high triglyceride level and reduction of MG length is quite innovative and we can assume that it is the first morphology change in meibomian shape that reflects the modification in the meibomium secretum. A change in meibomium component may modify its viscosity and cause the pressure to express meibomium from orifices to increase. Tao Yu et al. [26] identified an increase in resistance to outflow of meibomium in patients with type 2 DM due to some MG orifice obstruction. We believe that there are at least two factors to justify these changes. From one side, there is an increase in meibomium viscosity and from the other side, MG orifice obstruction due to keratinization related to pro-inflammatory process. This increase in pressure in the lumen of MGs could be an explanation for the increasing MG width, and this anatomical change can lead MGs to become atrophic.

BMI percentile was found to be a predictor of MG tortuosity and atrophy in pediatric patients [40] and also a risk for MGD in the adult population [41]. In our research, BMI was not associated with any parameter analyzed. Other colleagues [38] reported similar findings. In our population, BMI distribution was not representative because patients were classified only as normal and overweight without any patients in the obese percentile.

This study has several limitations; the sample we analyzed is small and was obtained from a single medical center. The incorporation of elderly subjects may also cloud the relationship between blood sample lipid abnormalities and MGD. In addition, our sample was representative only of female patients. In our center, male patients complaining of MGD symptoms were very few and many of them were taking anti-androgen medication or they were heavy smokers. Both were exclusion criteria for our study. To provide a correct representation of the population, the sample would need to be increased including also male patients. All data were obtained from a single visit and meibography was performed at only one point in time without prospective longitudinal studies, so it is difficult to ascertain whether MG changes in morphology were associated with DM status and/or change in hormone levels. In addition, the absence of control groups cannot allow to state any differences from MGD and non-MGD patients.

Overall, this study provides a starting point for further research. We believe that identification of clinical biomarkers is extremely useful for treating these patients, especially in those centers where meibography is not available. Cortisol, DHEAS, ASD and triglyceride levels showed interesting associations in our diabetic population. Regarding meibography in patients with type 2 DM, MG width enlargement seems to be the primum movens and its early detection can lead to an improvement of patient treatment. We cannot state that an early treatment can halt the progression of the disease and reduce the loss of MG area but it would be interesting to study the evolution of this parameter in longitudinal studies. Even if this study has several limitations, it is the first study to have reported some associations between MG parameters obtained by meibography and serum biomarkers. Future studies, perhaps including meibum lipid analysis and tear cytokine levels, may also further elucidate the connection between these parameters, MG architecture and function.

## Figures and Tables

**Table 1:** Marx Line Score [12].

Grade	Lower Lid Examination after Fluorescin Staining
0	ML runs along the conjunctival side of the Mos
1	Parts of the ML touch the Mos
2	ML runs through the Mos
3	ML runs along the skin side of the Mos

ML—Marx Line; Mos—Meibomian Orifices. The lower eyelid was further segmented into three portions: the outer third, middle third and inner third. The sum of the ML scores for the three portions of the lower lid was defined as the total score of each eye; therefore, the maximum possible ML score was 9.

**Table 2:** Demographics and clinical signs of studied patients.

Number of patients/eyes	18
Age (years)	67.76 ± 12.39 (32–81)
BMI (kg/m^2^)	29.05 ± 4.52 (21–36)
Tear break-up time (s)	2.94 ± 1.89 (0–7)
Schirmer test I (mm/5 min)	7.94 ± 5.97 (0–18)
Schirmer test II (mm/5 min)	5.29 ± 3.84 (0–14)
Marx line score	3.8 ± 2.7 (0–9)
Vbs score	1.29 ± 0.98 (0–3)
OSDI	32.55 ± 28.57 (0–90)

Values are presented as mean ± standard error of the mean. OSDI, ocular surface disease index; BMI, body mass index; vBS, Van Bijsterveld score.

**Table 3:** Results of regression analysis test between MG parameters and serum biomarkers.

	β	T	*p* Value
Glicemia (mg/dL) VS			
Meibobian length (mm/mm^2^)	−0.043	−0.087	0.933
Meibomian area (mm^2^)	0.097	0.254	0.805
Meibomian width top (mm/mm^2^)	−0.218	−0.309	0.764
Meibomian width centre (mm/mm^2^)	0.373	0.476	0.644
Meibomian width low (mm/mm^2^)	0.148	0.316	0.758
Testosterone (nmol/L) VS			
Meibomian length (mm/mm^2^)	−0.085	−0.178	0.863
Meibomian area (mm^2^)	0.342	0.861	0.410
Meibomian width top (mm/mm^2^)	−0.011	−0.019	0.985
Meibomian width centre (mm/mm^2^)	−0.137	−0.219	0.831
Meibomian width low (mm/mm^2^)	−0.051	−0.117	0.909
Trygliceridis (mg/dL) VS			
Meibomian length (mm/mm^2^)	0.078	0.242	0.813
Meibomian area (mm^2^)	0.264	1.002	0.336
Meibomian width top (mm/mm^2^)	−1.100	−2.420	0.277
Meibomian width centre (mm/mm^2^)	1.487	2.504	0.288
Meibomian width low (mm/mm^2^)	−0.705	−2.005	0.682
Total Cholesterol (mg/dL) VS			
Meibomian length (mm/mm^2^)	0.639	1.634	0.133
Meibomian area (mm^2^)	0.337	1.119	0.289
Meibomian width top (mm/mm^2^)	−1.024	−1.834	0.097
Meibomian width centre (mm/mm^2^)	1.558	2.512	0.204
Meibomian width low (mm/mm^2^)	−0.938	−2.536	0.115
HDL cholesterol (mg/dL) vs			
Meibomian length (mm/mm^2^)	0.529	1.350	0.207
Meibomian area (mm^2^)	−0.516	−1.709	0.118
Meibomian width top (mm/mm^2^)	−0.686	−1.227	0.248
Meibomian width centre (mm/mm^2^)	0.048	0.078	0.940
Meibomian width low (mm/mm^2^)	0.606	1.636	0.133
LDL Cholesterol (mg/dL) vs			
Meibomian length (mm/mm^2^)	0.388	0.737	0.494
Meibomian area (mm^2^)	0.105	0.246	0.815
Meibomian width top (mm/mm^2^)	0.170	0.215	0.838
Meibomian width centre (mm/mm^2^)	0.658	0.828	0.445
Meibomian width low (mm/mm^2^)	−0.347	−0.742	0.491
DHEAS (µg/dL) VS			
Meibomian length (mm/mm^2^)	−0.512	−1.520	0.154
Meibomian area (mm^2^)	0.050	0.183	0.858
Meibomian width top (mm/mm^2^)	1.377	2.884	0.921
Meibomian width centre (mm/mm^2^)	−1.415	−2.270	0.832
Meibomian width low (mm/mm^2^)	−0.052	−0.140	0.891
ASD (ng/mL) VS			
Meibomian length (mm/mm^2^)	0.478	2.174	0.045 *
Meibomian area (mm^2^)	0.051	0.166	0.871
Meibomian width top (mm/mm^2^)	0.505	0.950	0.361
Meibomian width centre (mm/mm^2^)	−0.598	−0.861	0.406
Meibomian width low (mm/mm^2^)	−0.041	−0.101	0.921
Cortisol (µg/dL) vs			
Meibomian length (mm/mm^2^)	0.510	1.321	0.211
Meibomian area (mm^2^)	−0.220	−0.695	0.500
Meibomian width top (mm/mm^2^)	−0.279	−0.510	0.619
Meibomian width centre (mm/mm^2^)	−0.024	−0.034	0.974
Meibomian width low (mm/mm^2^)	0.419	0.990	0.342

* statistically significant value.

## Data Availability

The data presented in this study are available on request from the corresponding author.
